# The clinical trial landscape in oncology and connectivity of somatic mutational profiles to targeted therapies

**DOI:** 10.1186/s40246-016-0061-7

**Published:** 2016-01-16

**Authors:** Sara E. Patterson, Rangjiao Liu, Cara M. Statz, Daniel Durkin, Anuradha Lakshminarayana, Susan M. Mockus

**Affiliations:** The Jackson Laboratory for Genomic Medicine, 10 Discovery Dr., Farmington, CT 06032 USA

**Keywords:** Cancer, Precision medicine, Actionability, Clinical trials, Curation

## Abstract

**Background:**

Precision medicine in oncology relies on rapid associations between patient-specific variations and targeted therapeutic efficacy. Due to the advancement of genomic analysis, a vast literature characterizing cancer-associated molecular aberrations and relative therapeutic relevance has been published. However, data are not uniformly reported or readily available, and accessing relevant information in a clinically acceptable time-frame is a daunting proposition, hampering connections between patients and appropriate therapeutic options. One important therapeutic avenue for oncology patients is through clinical trials. Accordingly, a global view into the availability of targeted clinical trials would provide insight into strengths and weaknesses and potentially enable research focus. However, data regarding the landscape of clinical trials in oncology is not readily available, and as a result, a comprehensive understanding of clinical trial availability is difficult.

**Results:**

To support clinical decision-making, we have developed a data loader and mapper that connects sequence information from oncology patients to data stored in an in-house database, the JAX Clinical Knowledgebase (JAX-CKB), which can be queried readily to access comprehensive data for clinical reporting via customized reporting queries. JAX-CKB functions as a repository to house expertly curated clinically relevant data surrounding our 358-gene panel, the JAX Cancer Treatment Profile (JAX CTP), and supports annotation of functional significance of molecular variants. Through queries of data housed in JAX-CKB, we have analyzed the landscape of clinical trials relevant to our 358-gene targeted sequencing panel to evaluate strengths and weaknesses in current molecular targeting in oncology. Through this analysis, we have identified patient indications, molecular aberrations, and targeted therapy classes that have strong or weak representation in clinical trials.

**Conclusions:**

Here, we describe the development and disseminate system methods for associating patient genomic sequence data with clinically relevant information, facilitating interpretation and providing a mechanism for informing therapeutic decision-making. Additionally, through customized queries, we have the capability to rapidly analyze the landscape of targeted therapies in clinical trials, enabling a unique view into current therapeutic availability in oncology.

## Introduction

The advent of the genomic era has provided clinicians and researchers the ability to analyze molecular data from patients and identify genetic variants that may have an impact on their clinical outcome and treatment options. Cancer research has additionally identified a myriad of genetic variations that impact protein function, the pathology of tumor cells, and potential response to targeted therapies. Connecting this information to clinical patient data is critical for the implementation of precision medicine. However, this information is vast and disparate, which hampers the ability to access potentially crucial information in a clinically acceptable time frame. Access to this data requires several key components: a structured and well-organized database for deposition of clinically relevant data, accurate manual curation of data with limited variability, accessibility of connections between data elements via well-defined relationships, and a system for routinely and automatically mapping clinical sample data to the database. A number of publicly available databases exist that catalog cancer-related genomic variations or that connect variations to potentially relevant therapies, but none complement the need for connecting patient aberrations to targeted therapy—either through clinical trials or approved drugs, while incorporating supporting efficacy information. For instance, the COSMIC database provides an invaluable catalog of cancer-related somatic genetic aberrations but does not assess relationships between those variants and therapies [1]. The My Cancer Genome database from Vanderbilt incorporates efficacy data for well-studied molecular aberrations that could prove useful in clinical interpretation [2]. However, the content is confined to a small variant list and is not routinely updated and as a result, the depth and breadth of the coverage of molecular targets and targeted therapies, as well as patient indications and clinical trials curated is limited, effectively hindering its utility. In addition to the scarcity of databases populated with comprehensive targeted oncology clinical data, a system that can directly link patient sequence data to clinical information is lacking, and thus, the speed at which these data can be related to targetable mutations in tumor samples is greatly reduced.

To enable this process, we have developed a clinical bioinformatics and curation pipeline that operates within a Clinical Laboratory Improvements Amendment (CLIA) and College of American Pathologists (CAP)-accredited environment, the JAX Clinical Genome Analytics (CGA) system. This system enables systematic identification and annotation of clinically relevant cancer variants and facilitates connections to therapeutic interventions. JAX-CGA comprises several components, including an automated data loader and mapper, which loads called variants from clinical samples and transforms them to Human Genome Variation Society (HGVS) nomenclature using Human Genome Organisation (HUGO) gene symbols and subsequently maps the variants to the database. In addition, our in-house curation database, the JAX Clinical Knowledgebase (JAX-CKB) enables dynamic curation of data connecting genetic variant to phenotype and protein effect, as well as therapeutic relevance and potential treatment approaches, which can be queried readily for clinical reporting. To facilitate interoperability between databases, the JAX-CKB utilizes standardized variant nomenclature and incorporates specific ontologies. HUGO, through the HUGO Gene Nomenclature Committee (HGNC), maintains a catalog of unique approved gene names, which we have incorporated into the JAX-CKB, reducing ambiguity and enabling interoperability between databases [3]. Additionally, the Human Genome Variation Society (HGVS) provides guidelines for the standardization of variant nomenclature, which are actively maintained and updated, facilitating unambiguous variant naming [4].

A fundamental challenge to precision medicine is the ability to easily connect a patient’s genetic variants with a therapeutic approach, which could include either FDA-approved targeted therapies or targeted therapies in recruiting clinical trials. Recent studies have demonstrated that linking patients to relevant targeted therapies based on genomic data has the potential to achieve higher clinical success relative to recruitment solely on histological subtype [5]. Leveraging the power of an integrated knowledgebase, we can identify opportunities for clinical intervention, including available clinical trials for targeted therapies. A comprehensive database can also provide a unique view into the landscape of molecular targets and therapies, uncovering potential opportunities for new research and development. In addition to supporting clinical reporting, the JAX-CKB allows visibility into deficiencies in the characterization of potentially actionable mutations and therapeutic interventions, exposing opportunities for research that have the potential to advance cancer treatment. Here, we detail and share the processes by which we map patient data to the knowledgebase and provide a detailed view of the clinical trial landscape in solid tumors.

## Results and discussion

**Fig. 1:**
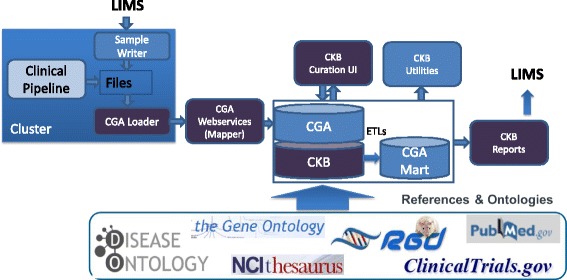
Flow of data through the clinical pipeline. Patient sample sequence data from the clinical genome analytics (CGA) pipeline is integrated with sample meta-data from the Laboratory Information Management System (LIMS), and called variants are mapped to the JAX-Clinical Knowledgebase (JAX-CKB) database. The JAX-CKB incorporates information populated from various incorporated ontologies and databases, and via the curation User Interface (UI), and is maintained using the CKB-Utilities tools. Using information from the JAX-CGA/CKB and the CKB reports, tools are used to generate datasets to enable clinical report generation

The organization of the CGA system (Fig. 1) incorporates several components, including the bioinformatics pipeline, the data loader and mapper, the JAX Clinical Knowledgebase, and reporting tools. The bioinformatics pipeline portion of the CGA system, including its validation, is discussed elsewhere [6].

The flow of data through the CGA-CKB system begins with the transformation and automated mapping of patient variants to JAX-CKB. Mapped variants are then filtered for actionability, which is defined as those with a related FDA-approved therapy or targeted therapy in clinical trials. Filtered actionable variants are then linked to treatment approaches and relevant recruiting clinical trials using unique clinical reporting tools through drug class or therapy, as well as by molecular criteria. This process is facilitated by the design of the JAX-CKB database, which incorporates connectivity of various database attributes, through the use of standard language and ontologies. The user interface allows for annotation of the most current data surrounding genetic variants and targeted therapies, on top of a system that incorporates standard ontologies and controlled vocabularies. This data is then readily accessible via reporting and analysis queries for both patient clinical reporting applications and for comprehensive analysis of data, including data regarding clinical trials. To enable both clinical reporting and analysis of clinically relevant data surrounding the JAX-CTP-targeted gene panel, we have implemented a unique framework for managing and connecting patient molecular data to efficacy information and relevant treatment approaches in real-time.

### Data loader and mapper

The data loader and mapper play a critical role in routine processing of large scale variation results from the CGA pipeline. The roles of the data loader and mapper are to upload genomic sequence data and integrate that sequence data with sample meta-data and to automatically map patient variants to JAX-CKB, enabling further queries for clinical interpretation.

**Fig. 2:**
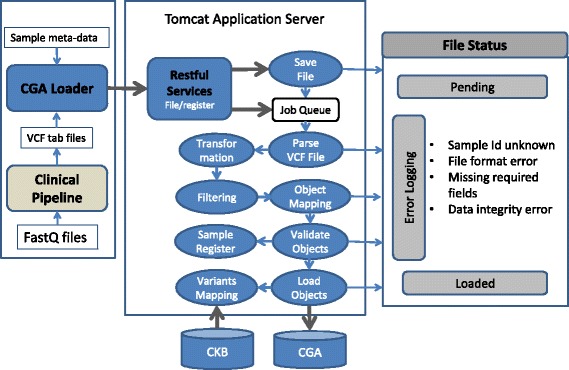
Automated data loading process. The data loader scans the file system hourly to find any new runs from the clinical pipeline and submits the variant call files (VCF) along with their sample meta-data to a web service. The web service manages the data loading jobs in a queue, to transform, filter, and upload the datasets to our CGA database and map the variants to the JAX-CKB

The CGA loader (Fig. 2) runs as a daemon program, which intermittently scans the file system in a cluster server searching for new runs from the clinical analytics (CGA) pipeline. New runs consist of either analysis results of a new sample or repeat analysis results from a previously mapped sample. The CGA loader program additionally extracts sample meta-data, such as patient diagnosis, from our Laboratory Information Management System (LIMS) and submits these data along with the variant call format (VCF) files to a Restful web service.

The web service, running in a Tomcat application server, saves all data files in the database with an initial “pending” file status and manages the workflow of all computational jobs in a queue. The format of each data file is defined as a template in the database allowing an integrated Java program, using Reflection, to dynamically parse, transform, and map file columns to database tables and columns. During this workflow, additional filtering or validation steps can be integrated for quality or biological reasons. If an error occurs in the loading process, the file status will be updated as “error” and a tracking message will be logged. Following successful completion of a job, the file status will be updated as “loaded.”

**Fig. 3:**
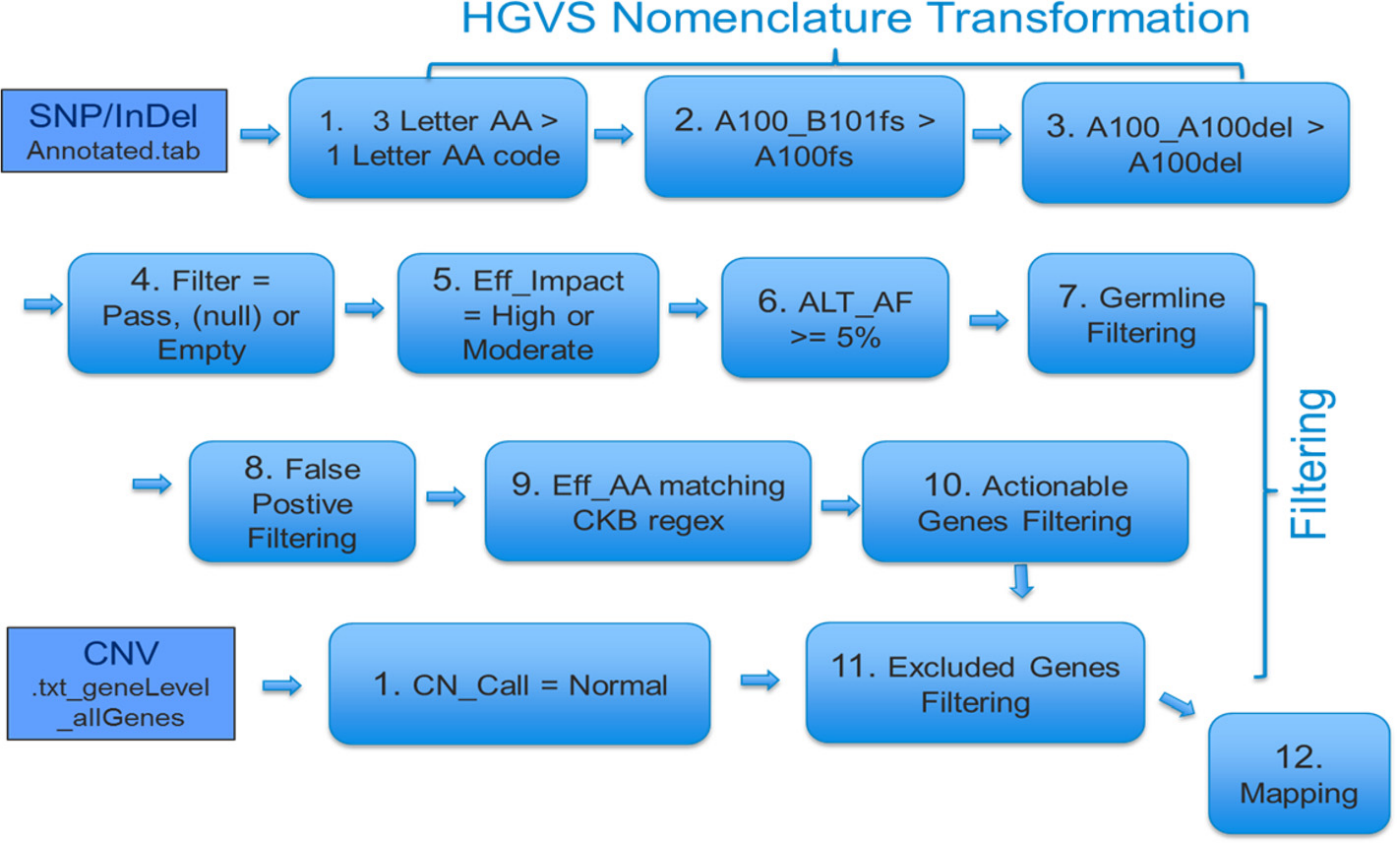
Automated variant mapping process. The genomic datasets are transformed to standard Human Genome Variation Society (HGVS) nomenclature, using a standardized Regular Expressions (regex) vocabulary incorporated into the JAX-CKB. This enables mapping and identification of clinically meaningful variants in a scalable and reproducible manner. The variant mapping program also includes several filtering steps, including removal of low-impact variants, those that did not pass metrics, and those that are likely germline or false-positive, to ensure reporting of only high-quality somatic variations

The Mapper (Fig. 3) is the nickname of the web service that manages the automated data loading and mapping jobs. During the data loading workflow, sequence variants, which include point mutations or small insertions or deletions (InDels), are transformed to standard Human Genome Variation Society (HGVS) nomenclature [4], which is consistent with the regular expression (regex) vocabulary of variants in the JAX-CKB. For example, the SnpEff and SnpSift programs annotate an amino acid from nucleotide sequence information using a three-letter amino acid code (e.g., “Gly” for Glycine). This three-letter code is then transformed to the single letter code for that amino acid, using the appropriate syntax (e.g., “G” for “Gly”). For example, p.Gly749Glu/c.2246G > A will be transformed to “G749E,” and the frameshift Arg34_Val35fs/c.102_103insG will be transformed to “R34fs.” Subsequent to transformation, several filtering steps have been implemented to ensure the use of only high-quality somatic variations and reduce false-positives. Filters include removal of variants with low coverage, variants with allele frequencies that do not meet minimum requirements for sensitivity, low impact variants including silent mutations, variants that are likely germline, and those that are likely false-positive or outside of coding regions. Simultaneously, Copy Number Variation (CNV) files are filtered to remove genes with fewer than six copies, based on the analytical validation of the assay [6]. Following filtering, CNV files are merged with the filtered VCFs. The analytics program will then automatically recognize and map the variants in the JAX-CKB, enabled by the transformation to the HGVS syntax used by JAX-CKB.

This process allows for direct connectivity between clinically actionable somatic variants present in patient tumor samples with applicable clinical data.

### Database queries and clinical reporting tools

While compiling and organizing clinically relevant data has utility, the power to utilize these data lies in the ability to easily access and leverage complex data to inform clinical decision-making. To that end, we have designed and implemented several database queries, which both facilitate reporting and enable unique views into the data for analysis and quality assurance.

Following the mapping of samples to the JAX-CKB, mapped variants are filtered and displayed in a JAX-CKB reporting application. From the sample landing page, a user can select the relevant sample and launch queries directly from the sample results, allowing the user to tailor the queries to sample-specific molecular aberrations while providing traceability. Several queries have been built to generate result sets that support clinical reporting. The queries are designed to run based on all clinically relevant variants in a single sample simultaneously; however, the search page for each query incorporates a selection tool from which a user can deselect any variant in the sample to exclude as search parameters. This enables the user to run the queries on only variants that have clinical relevance, as well as limiting query results from samples with many actionable variants to produce multiple, more manageable, datasets. To retrieve the list of targeted therapies associated with sample-specific variants, we have designed a query that utilizes the associated drug class or individual therapy treatment approaches specific to variants to retrieve all drugs assigned to those treatment approaches. For example, the variant KIT D816V has the associated treatment approach “KIT Inhibitors.” There are currently 31 drugs curated into JAX-CKB that have been assigned the drug class “KIT Inhibitors.” If present in a mapped sample in CKB, launching the drug query retrieves the drug class treatment approach for KIT D816V and from this retrieves the list of drugs associated with this drug class. In addition, there are three separate queries that leverage different aspects of curated efficacy evidence. The approval status query retrieves data from the “approval status” attribute of curated efficacy evidence for all drugs related to treatment approaches for given variants. This enables identification of targeted therapies that are FDA-approved versus investigational therapies, related to a treatment approach for the patient variant(s). The drug resistance query retrieves efficacy evidence entries for all molecular profile responses with response type of “resistant” that contains the same parent gene of sample-specific variants. For example, for a sample containing a KRAS G12C variant, the resistance query would retrieve all resistance efficacy evidence associated with any molecular profile response containing a variant in KRAS. This would include the efficacy evidence related to KRAS G12C, as well as category variants “KRAS G12X” and closely related variants KRAS G12D and KRAS G13C. Relating to parent gene, rather than specific variant, enables visibility into all potential resistance mechanisms without requiring duplication of data in CKB. A third query pulls all evidence with sensitive response type related to the treatment approach for patient variants, to support rationale for therapies included on patient reports.

#### Clinical trial query

Clinical trials function as an avenue toward clinical intervention and constitute an important component of the therapeutic landscape for oncology patients. Thus, we have incorporated the identification of clinical trials relevant to patient variants into clinical reporting to provide multiple potential options for clinicians to direct patient treatment. Clinical trials can be relevant to patient variants via inclusion of drugs contained in variant treatment approaches, or through molecular inclusion criteria. To retrieve a list of open clinical trials related to sample variants, we have implemented a complex query that retrieves clinical trials both for drugs in treatment approaches and those that contain molecular criteria for the parent gene associated with sample variants, irrespective of therapy. Clinical trials are queried first by drugs contained in variant treatment approaches, and for resulting trials containing those drugs, data is retrieved and displayed including NCTID, sponsor, title, and therapy, as well as a column including the variant for which the trial was pulled. Clinical trials are also queried on parent gene, excluding all trials with a requirement for wild-type. Clinical trials can be further restricted through the selection of Disease Ontology terms, related to patient indication. The Disease Ontology (DO) is a robust, regularly maintained, ontology of standardized disease terms, each backed by individual unique identifiers (DOIDs), which facilitates precise mapping to patient diagnosis [7]. When selected, returned trials will include only those that are related to the selected DOID and all child terms. This process is enabled by the integration of the DO tree in clinical trial curation, which can be employed for retrieval of only those trials relevant to the patient’s indication.

### Clinical trial landscape

Outside of CKB, there are currently no publicly available comprehensive databases that curate information on molecular eligibility for clinical trials. Access of clinical trials recruiting on molecular criteria is not readily accessible through clinical trial registries, such as clinicaltrials.gov, which is primarily the result of a lack of standardization of nomenclature and syntax for molecular eligibility criteria in clinical trial registries [8]. One significant potential outcome of this is an inadequate representation of potentially relevant clinical trials for clinicians performing a cursory keyword search. In addition, the lack of molecular criteria accessibility likely increases the time necessary for identifying potentially relevant trials, as a clinician must sort through the trial record to identify molecular inclusion criteria. To expedite retrieval of relevant clinical trials and streamline the path toward clinical intervention based on molecular variant, we have incorporated manual curation of molecular eligibility into the JAX-CKB database to easily connect patients with trials that are relevant to findings from next-generation sequencing.

In addition to providing a path to intervention in clinical reporting, the organization and design of the JAX-CKB (discussed below) allows us to readily analyze the current landscape of clinical trials. This analysis of the relative number of clinical trials for drug class or molecular criteria enables a view into the strengths and weaknesses in targeting actionable variants in clinical trials. Previous analysis of the landscape of cancer clinical trials in the USA, in the absence of a structured curated database, has required significant effort using data extracted manually via keyword search of clinicaltrials.gov [9]. The organization of data elements curated into the JAX-CKB database facilitates rapid analysis of data from clinical trials through customized queries of the database. Using these queries, we have analyzed the landscape of trials restricted to those currently open (defined as those with the recruitment status of “not yet recruiting,” “recruiting,” or “available”) related to our 358-gene targeted panel.

**Fig. 4:**
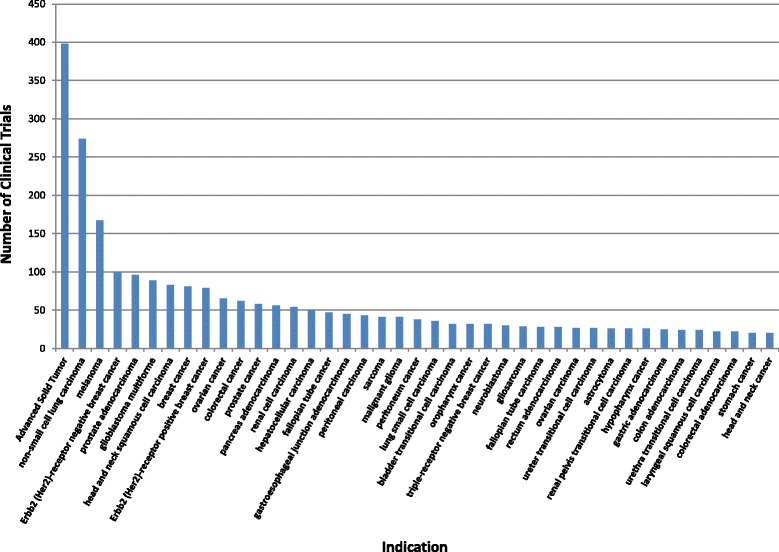
Analysis of JAX-CKB curated clinical trials by solid tumor indication. Disease Ontology terms selected as patient indication for greater than 20 clinical trials plotted relative to the number of open clinical trials recruiting on each indication

When broken down by patient indication (Fig. 4), 19.1 % of open trials curated into the JAX-CKB are recruiting patients with advanced solid tumors with unspecified histology (398/2058; Fig. 4). This comprises the largest proportion of curated clinical trials and likely represents early stage trials for therapeutics that have not demonstrated clinical utility in specific tumor types. Other well-studied indications are highly represented in clinical trials (Fig. 4), particularly those for which a recurring molecular target has been identified. These include non-small cell lung cancer (13.23 %; 274/2058), melanoma (8.0 %; 167/2058), ERBB2 (Her2)-receptor negative breast cancer (4.8 %; 100/2058), prostate adenocarcinoma (4.7 %; 96/2058), glioblastoma multiforme (4.3 %; 89/2058), and head and neck squamous cell carcinoma (4.0 %; 83/2058) (Fig. 4). There are 96 indications present as inclusion criteria in only a single open clinical trial for a targeted therapy, representing an unmet need for these patients. These indications include rare cancers, such as chordoma (NCT01407198) and hemangiopericytoma (NCT01396408), which have very little data surrounding targeted therapeutic efficacy, and accordingly very few promising treatment options [10, 11].

**Fig. 5:**
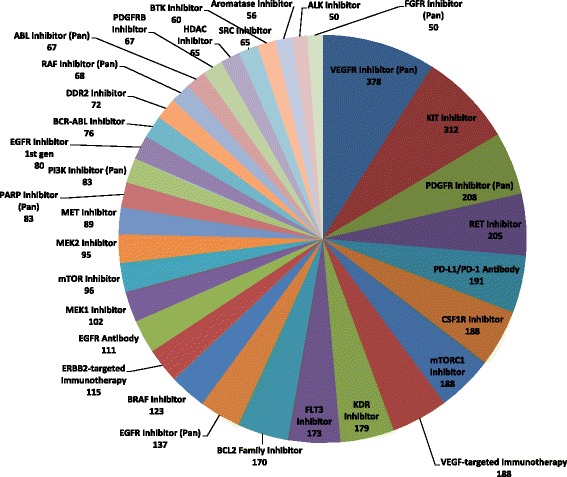
Analysis of open clinical trials related to drug class. Chart displays the number of clinical trials investigating targeted drugs within specified drug classes, for all drug classes represented in greater than 50 open clinical trials

Independent of histology, patients may enter clinical trials based on molecular eligibility, either through a molecular aberration that is targeted by an agent in the trial, or a trial that is directly recruiting on the presence of a specific molecular criterion, irrespective if the investigated therapy has demonstrated a connection to the molecular target. Drug classes are a proxy for treatment approach, and identifying the number of clinical trials investigating drugs within specific drug classes provides insight into the availability of targeted therapies for molecular aberrations. In an analysis of the most frequent drug classes containing drugs currently under investigation in open clinical trials, we have determined that Pan-VEGFR Inhibitors (378/2168; 17.4 %), KIT Inhibitors (312/2168; 14.4 %), Pan-PDGFR Inhibitors (208/2168; 9.6 %), and RET Inhibitors (205/2168; 9.5 %) are most highly represented (Fig. 5). This suggests that patients with activating mutations in KDR (VEGFR2), KIT, PDGFR, and RET, depending on indication, may have several options for potential therapeutic intervention through recruiting clinical trials. In contrast, the drug classes DNMT1 inhibitors, EZH2 inhibitors, and FGFR3 antibody are among those represented in a single open clinical trial, which suggests that patients harboring genetic aberrations targeted by these drug classes may have fewer options.

**Fig. 6:**
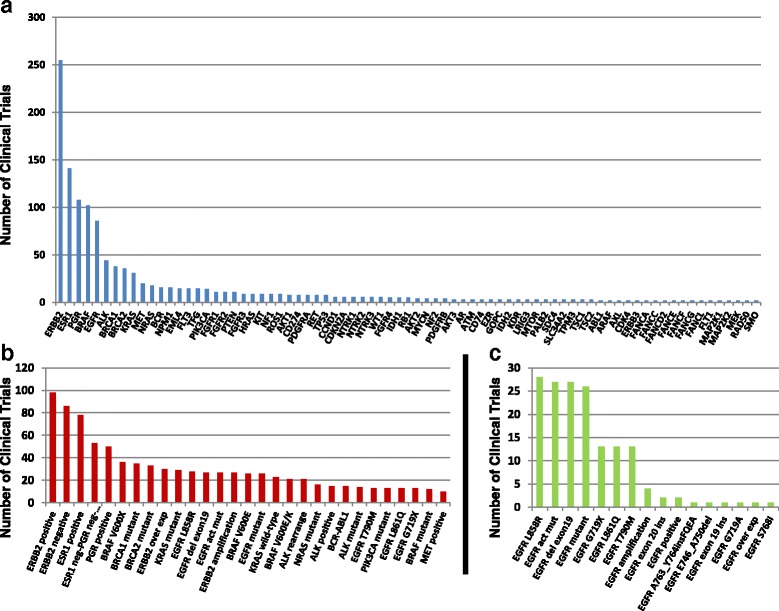
Analysis of number of open clinical trials relative to gene and gene variant as molecular criteria. **a** Combined total of clinical trials with eligibility criteria related to individual genes. Graph displays genes that are represented by molecular eligibility criteria in two or greater clinical trials. **b** Number of clinical trials for the top specific or category molecular criteria represented in clinical trials, limited to those in 10 or more clinical trials. **c** Number of clinical trials for each type of EGFR molecular criteria present in open clinical trials

Clinical trials may also recruit patients based directly on molecular eligibility, which may be restricted to specific molecular aberrations, or a wider category of molecular aberrations. For instance, a patient with an exon 19 deletion in EGFR could potentially enroll in clinical trials recruiting patients with “EGFR mutations,” “EGFR activating mutations,” “EGFR exon 19 deletions,” or the specific deletion harbored by this patient. Currently, the top 5 molecular criteria in open clinical trials are related to the expression of various hormone and growth factor receptors including ERBB2, PGR, and ESR1 (Fig. 6), which is likely related to their long-standing clinical association with breast cancer subtyping and therapeutic relevance [12]. Mutations in BRAF V600 have demonstrated clinical utility as targetable aberrations [13] and are highly represented as molecular eligibility criteria in clinical trials, with 36 open trials recruiting on BRAF V600 mutations at the time of this manuscript (Fig. 6b). BRCA1 and BRCA2 mutations are also frequently included as molecular eligibility criteria, with 35 and 33 open trials recruiting on these variants, respectively (Fig. 6b).

EGFR aberrations are a highly represented target for investigational therapies in clinical trials. EGFR is the fifth most common gene associated with molecular inclusion criteria for open clinical trials curated into CKB, with 86 clinical trials including EGFR-related molecular inclusion criteria (Fig. 6a). Most of these trials are related to the well-characterized EGFR L858R and exon 19 deletion mutations (Fig. 6c), which have demonstrated sensitivity to EGFR tyrosine kinase inhibitors (TKIs) [14]. In contrast, EGFR exon 20 insertion mutations demonstrate de novo resistance to EGFR TKIs and have few options for clinical intervention [15, 16]. At the time of this manuscript, EGFR exon 20 insertions are included as eligibility criteria for two open clinical trials. Both of these trials are investigating the HSP90 inhibitor AUY922 and are recruiting either specifically on exon 20 insertions or for EGFR mutations, including exon 20 insertions. HSP90 inhibitors have not demonstrated full clinical utility in the context of EGFR mutations but have preclinical support [17]. In this context, HSP90 inhibitors do not meet the stringency for a JAX-CKB treatment approach, and thus would not be an entry point for clinical trial association for patients with EGFR exon 20 insertion mutations. Therefore, the inclusion of molecular criteria in this instance represents a mechanism for therapeutic intervention that would not otherwise be available.

### The JAX clinical knowledgebase and curation user interface

**Fig. 7:**
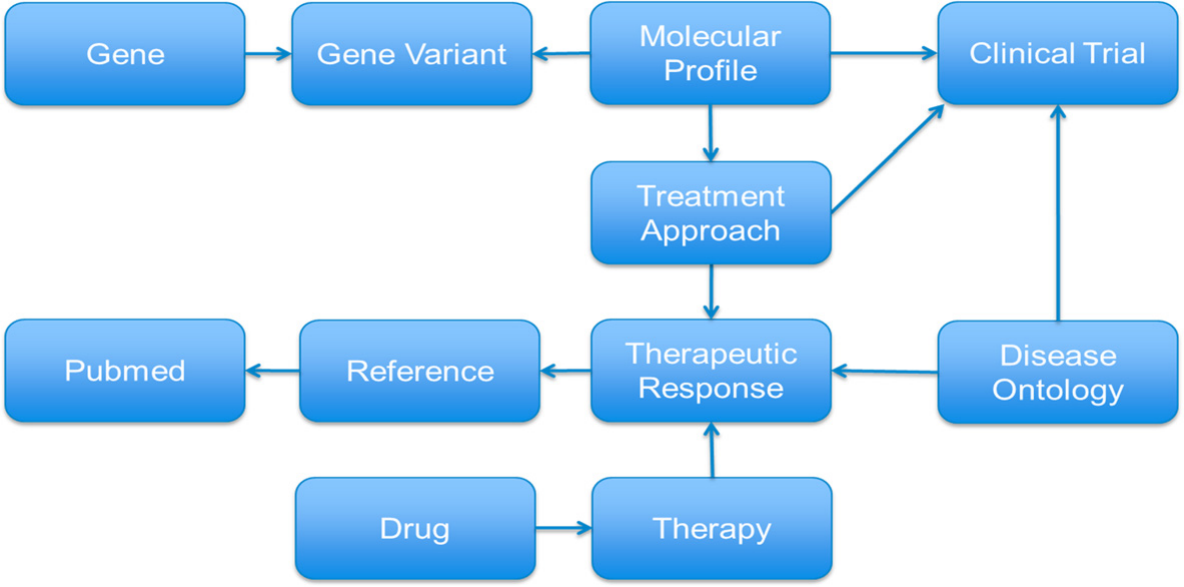
Connections between various data elements in the JAX Clinical Knowledgebase (JAX-CKB). Molecular profiles consist of one of more molecular entities, linked to gene variants and parent genes. Molecular profiles are assigned relevant treatment approaches based on available data from the literature on efficacy, as a therapeutic response. The therapeutic response additionally takes into account patient indication, which is represented by an integrated disease ontology. Treatment approaches contain therapies, which consist of a single drug or combination of drugs. These drugs can be either FDA-approved, or under investigation in clinical trials, which are additionally curated into the JAX-CKB

A critical component to accessing data for reporting or analysis is a comprehensive structured database. Thus, we have designed and implemented an in-house database to support clinical reporting and analysis of clinically relevant data. The JAX-CKB serves as a repository for data regarding oncology-relevant genetic variants and therapies and incorporates interconnectivity between protein effect, therapeutic efficacy, treatment approaches, and recruiting clinical trials. CKB features a structured database framework for managing clinical and biological data, into which clinical data content is entered through a curation user interface (Fig. 7). Data is dynamically curated by experts to populate comprehensive content related to oncology, to support clinical reporting, and to maintain the most up-to-date information on therapies, response, and relevant clinical trials. While JAX-CKB content is currently tailored toward solid tumor clinical reporting, the framework of the JAX-CKB is versatile and can support additional applications. The organization of the JAX-CKB is essential to its utility in supporting clinical reporting and includes several key elements (Fig. 3), which are outlined below.

#### Data elements

The JAX-CKB database incorporates several data elements, which are connected in various ways in curation of cancer-related data.

#### Genes and gene variants

To support standardization and mapping of genetic variants to JAX-CKB and facilitate interoperability with other databases, we have incorporated HUGO Gene Nomenclature Committee (HGNC) nomenclature through the integration of approved symbols and gene names from the NCBI Gene database into the JAX-CKB [3, 18]. By incorporating gene names from the Gene database, genes are assigned approved symbols, removing variability, and are additionally backed by unique numerical gene identifiers (NCBI GeneID). Although our current JAX Cancer Treatment Panel (JAX CTP) is limited to 358 cancer-related genes, the JAX-CKB incorporates all known annotated human genes. This supports the rapid pace of advancement in cancer research, as well as allowing for the flexibility to support additional assays, which would include sequencing assays for other disease types.

Naming of genetic variants in the JAX-CKB utilizes a Regular Expression (regex) system that conforms to Human Genome Variation Society (HGVS) nomenclature guidelines, restricting the entry of genetic variants to a predefined syntax and enabling mapping of variants to JAX-CKB. HGVS maintains standards for variant naming, which serves to eliminate ambiguity and ensure precision when reporting data related to genetic variants [4]. Conforming to these standards additionally provides a mechanism to promote interoperability between databases. Each variant type is assigned a specified syntax. For example, a mutation resulting in a single amino acid change, such as the gene variant KRAS G12C, would follow the syntax [ACDEFGHIKLMNPQRSTVWY][0-9] + [ACDEFGHIKLMNPQRSTVWY]. The regex syntax is periodically updated to maintain compliance with HGVS guidelines. The JAX-CKB additionally incorporates controlled vocabularies regarding protein effect, to annotate functional relevance of specific variants. This aids in the interpretation of actionability and provides a further mechanism for filtering gene variants. Gene variants are linked to a parent gene and can include any alteration to the gene, supporting multi-omic data curation. Examples of variants include mutations, changes to gene copy number, and expression changes. In addition, JAX-CKB supports annotation of category variants (e.g., activating mutation, inactivating mutation), which allows molecular profile responses and clinical trials to be curated to conditions where they are applicable to multiple or unknown/unspecified aberrations. For example, the category variant “EGFR activating mutation” represents any activating mutation in EGFR. There are also less broad category variants, such as “EGFR del exon 19,” which represents a non-specified deletion within exon 19 of the EGFR gene. These variants are also subject to regex syntax and must meet predefined specifications. The inclusion of category variants enables capture of information that is relevant to bins of variants, rather than individual variants, or in conditions where the applicable variants are not specified.

Within the Gene Variant Curation User Interface (UI), there are multiple fields for data capture, each utilizing their own specific controlled vocabularies to minimize variability and allow for efficient analysis of curated information. The curation user interface allows users to capture information around genetic variants related to amino acid change, mutational impact to the protein (e.g., missense, deletion, and frameshift), and functional effect on the protein (e.g., gain-of function, and loss-of-function). In addition, gene variants can be linked to their impact on biological pathways, through the integration of a human pathway ontology from the Rat Genome Database [19]. The gene variant UI also incorporates a free-text annotation field for capture of specific information on the relationship of the variant to oncogenic transformation and protein function, which is supported by curated references from primary literature.

#### Molecular profiles

A molecular profile in the JAX-CKB contains one or more genetic aberrations, which can include mutations, such as single nucleotide polymorphisms (SNPs), frameshifts, insertions, and deletions, as well as gene fusions, copy number variations and/or changes in expression levels. The flexibility of the molecular profile and the ability to build complex molecular profiles allow for the curation of therapeutic efficacy to multiple aberrations simultaneously, rather than each variant in isolation. For example, a molecular profile may contain a mutation, such as the EGFR activating mutation EGFR L858R, as well as a copy number variation, such as amplification of the MET gene. MET amplification is a recognized mechanism for resistance to EGFR tyrosine kinase inhibitor therapy [20–22], and the ability to create a complex profile enables the curation of resistance data to the appropriate complex molecular conditions.

Current research supports the idea that targeted treatment of tumor driver mutations alone often leads to the upregulation of compensatory mechanisms and/or acquisition of secondary resistance mutations and that utilizing a multi-target approach may be beneficial in reducing the potential for resistance [23]. For example, the ability to assign treatment approaches and curate efficacy evidence to multiple aberrations simultaneously enables JAX-CKB and downstream clinical reporting to stay on-pace with clinical progress in the use of combination therapies in complex multi-omic conditions.

#### Drugs and therapies

A key component to capture response data related to molecular targets is a comprehensive database of relevant drugs, which are incorporated as data elements into JAX-CKB. Attributes associated with drugs include drug name, trade name, and synonyms, in which each are required to be unique terms within a drug record, and among other records, to effectively eliminate redundancy. CKB currently contains 1108 unique targeted therapies relevant to treatment approaches or clinical trials for variants related to our 358-gene panel. When appropriate, drugs are assigned a “Drug Class,” as a selectable field within the Therapy UI using an in-house controlled vocabulary. Drug class represents a rational group of drugs with a unified target, such as “PIK3CA Inhibitor” for drugs that target PIK3CA. These drug classes are then utilized as “Treatment Approaches” for a molecular profile, which provides a link between molecular aberrations and relevant targeted therapies.

Therapies, similar to molecular profiles, are comprised of one or more drugs. The ability to build complex therapies supports the therapeutic trend toward using targeted therapies in combination with other targeted therapies or chemotherapies and enables precision and accuracy in efficacy curation.

#### Treatment approaches

The current utility of CKB is to support clinical reporting for our JAX CTP panel. To this end, we have incorporated the selection of potential treatment approaches for molecular profiles, which designates a relationship between a selected molecular profile and either a drug class or individual therapy. Treatment approaches are selected following extensive review of the literature supporting preclinical and/or clinical efficacy, which is additionally curated via molecular profile response. For example, the gene variant PIK3CA H1047R is a gain-of-function variant in PIK3CA [24]. Due to several lines of evidence, PIK3CA H1047R has been assigned the drug class treatment approaches “mTOR inhibitors,” “PI3K inhibitors,” and “AKT inhibitors.” Each of these drug classes is linked to a milieu of drugs with the target mTOR, PI3K, and/or AKT, respectively. These treatment approaches are used in the reporting queries to identify relevant therapies and clinical trials.

#### Molecular profile response and efficacy evidence

An essential component of a clinical knowledgebase is the inclusion of evidence from primary literature sources supporting therapeutic efficacy in the context of specific molecular variants. The “Molecular Profile Response” element of the JAX-CKB represents the relationship between a molecular profile and indication, therapy, and related response type. The patient indication attribute integrates the cancer terms from the Disease Ontology [7], which are backed by unique identifiers (DOID) and represented in a navigable tree.

Associated with the molecular profile response is efficacy evidence, which is the point of entry for literature-based data supporting the molecular profile response. A molecular profile response can be associated with one or more lines of efficacy evidence. Efficacy evidence additionally includes information regarding the approval status of the associated therapy in relation to the curated evidence, using a controlled vocabulary to designate whether the study was preclinical, phase I, phase Ib/II, phase III, or FDA-approved, or if the therapy-molecular profile association is in National Comprehensive Cancer Network (NCCN), FDA, or European Society for Medical Oncology (ESMO) Guidelines. Curation of FDA-approval studies links tumor type to approval status, allowing for simultaneous identification of targeted therapies that have achieved FDA-approval in a patient indication. Efficacy evidence can be categorized, using controlled vocabulary, as actionable, which is characterized as data relating the efficacy of the selected therapy in the context of the selected profile response and is prioritized based on an in-house 25-tier ranking system. Additionally, the JAX-CKB supports the entry of prognostic and diagnostic evidence as well as emerging evidence, which supports potential emerging therapeutic targets.

#### Clinical trials

To facilitate the inclusion of relevant clinical trials in patient reporting to provide an additional potential avenue for therapeutic intervention, we have incorporated clinical trial content into the JAX-CKB. This additionally provides a unique mechanism for the analysis of clinical trial data, which is cumbersome through direct extraction from clinical trial databases. For the import of clinical trials into CKB, we have built a utility that searches clinical trials from clinicaltrials.gov using a predefined list of targeted therapies, restricted to trials in the USA or Canada in “cancer” that are “open,” which are defined as those trials that have a recruitment status of “recruiting,” “not yet recruiting,” or “available.” Returned trials are matched against those in the JAX-CKB to produce a unique list of new clinical trials. Information including NCTID, title, and recruitment status is imported, and remaining information from the trial record that cannot be automatically retrieved from clinicaltrials.gov is manually curated. The NCTID serves as a unique identifier for each clinical trial. The elements in the clinical trial user interface enable connectivity to molecular aberrations in two ways. First, an intervention attribute enables curation of therapies being tested in the clinical trial. These therapies are selected from a drop-down list of therapies that is populated from the therapy table. The drugs contained in those therapies can then be linked to a treatment approach via a drug class or individual therapy. Additionally, clinical trial curation integrates molecular eligibility criteria, so trials recruiting based on the presence or absence of specific molecular criteria can be easily identified. Therefore, a patient sample with an EGFR mutation would be linked to a trial testing erlotinib that is recruiting on EGFR activating mutations both by the targeted therapy and the required molecular criteria.

To ensure that we have the most up-to-date information in the JAX-CKB regarding clinical trial status, we have built a cron job that performs a nightly search of clinicaltrials.gov for any change to recruitment status for trials in the JAX-CKB and automatically updates any changes to the database. Thus, only clinical trials that have open recruitment statuses are included on clinical reports.

The connectivity of data in JAX-CKB and the associated reporting tools enables a direct connection between molecular aberration and targeted therapy and connects patients to trials investigating a relevant targeted therapy, but which may not be specifically recruiting on a molecular aberration. This may have the unintentional, beneficial consequence of increasing success and development of targeted agents by prescreening patients outside of the clinical trial protocol for the presence of relevant genomic targets.

## Conclusions

Progress in patient treatment in oncology is dependent on both a comprehensive understanding of the relationships between molecular variants in tumors and efficacy, and the power to access clinically relevant information in a timely manner. Here, we share methods and demonstrate the implementation of a data mapper and loader and queries for rapid retrieval of data related to clinical efficacy to inform clinical interpretation of molecular aberrations, supported by the structured and organized design of the JAX Clinical Knowledgebase. In addition, global analysis of the clinical trial landscape provides insight into the types of targeted agents and molecular targets that are focal points, as well as those that could be served by additional investigation.

## Methods

### JAX-CKB mapper and JAX-CKB schema

The following versions of the JAX-CKB mapper and database were used in analysis:

- JAX-CKB mapper: Version 1.05.
- JAX-CKB Schema: Version 1.4. JAX-CKB; 81 Tables.

### Data analysis

For data analysis, data was retrieved from the stage version of the JAX-CKB database using SQL queries. Due to the dynamic nature of the data in the production database due to continual curation, the stage database, which is not subject to continual update, was used to eliminate variability in the data. The stage database was last updated on September 18, 2015, from a snapshot of the production version of the JAX-CKB database.

### Clinical trial recruitment status updating

A nightly cron job is implemented to query https://clinicaltrials.gov/ct2/results. It parses the HTML contents to obtain the trials that match the search criteria [cancer-related clinical trials in the United States and Canada] and check the recruitment status of all existing trials in the JAX-CKB database. Following comparison with the recruitment status of clinical trials in the JAX-CKB database, it updates the database and sends an email notification to data curators if any recruitment status is changed.

## Abbreviations

CKB: Clinical Knowledgebase
CGA: Clinical Genome Analytics
CTP: Cancer Treatment Profile
DO: Disease Ontology
HGNC: HUGO Gene Nomenclature Committee
HGVS: Human Genome Variation Society
HUGO: Human Genome Organisation
regex: Regular Expression
VCF: variant call format
